# Anesthesia using remimazolam during coronary artery bypass surgery in a patient with decreased left ventricular function

**DOI:** 10.1002/ccr3.7970

**Published:** 2023-09-25

**Authors:** Shingo Narumi, Yusuke Ishida, Sae Igarashi, Shunya Sekiguchi, Aya Kawachi, Mikiko Tomino

**Affiliations:** ^1^ Department of Anesthesiology Tokyo Medical University Tokyo Japan

**Keywords:** angina, cardiac output, coronary artery bypass graft (CABG), ejection fraction, remimazolam

## Abstract

**Key Clinical Message:**

Remimazolam is a new benzodiazepine sedative and has the characteristic of causing minimal effects on circulation. This case indicates that it can be considered as an option for anesthesia management of patients with decreased cardiac function.

**Abstract:**

Some patients who undergo cardiac surgery have reduced cardiac function, which can often make anesthesia management difficult owing to severe hypotension at the time of anesthesia induction. Therefore, it is important to select drugs that cause minimal circulatory depression. On the other hand, in 2020, the use of remimazolam, a short‐acting benzodiazepine sedative, was approved in Japan, and reports of its use in various patients have been increasing. This drug has the characteristic of causing minimal effects on circulation. We here report the safe use of remimazolam in the anesthesia management of a patient with decreased cardiac function who was diagnosed as having angina pectoris. The patient was a 73‐year‐old man scheduled for coronary artery bypass graft (CABG) surgery. Remimazolam was used for sedation purposes during anesthesia induction. During surgery, there were no significant hemodynamic changes and the patient remained in stable cardiovascular condition. Our present case indicates that remimazolam can be considered as an option for anesthesia management in CABG for patients with decreased cardiac function.

## Graphical abstract

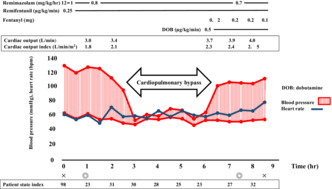

## 1 BACKGROUND

In the management of anesthesia during cardiac surgery, it is important to stably maintain the patient's cardiovascular function throughout the operation. Therefore, it is necessary to carefully evaluate the patient's cardiovascular function before surgery, monitor their general condition, and choose sedatives and analgesics carefully during anesthesia management. On the other hand, remimazolam, an ultra short‐acting benzodiazepine, has been available for use in Japan since 2020.^1^ This drug has a short half‐life and causes minimal circulatory depression,^2^ making it a suitable sedative for patients with decreased cardiac function. In this report, we used remimazolam safely in the anesthesia management of coronary artery bypass graft (CABG) surgery, and present our findings. Written informed consent was obtained from the patient to publish this case report.

## 2 CASE PRESENTATION

The patient was a 73‐year‐old man with a height of 170 cm and weight of 58 kg. He had a history of diabetes and chronic kidney disease and was being followed up by the nephrology department of our hospital. This time, he was admitted to the coronary care unit for fluid control owing to acute exacerbation of chronic kidney disease and a diagnosis of congestive heart failure. Electrocardiography displayed flat T‐waves in the inferior leads (II, III, and aVF leads), and negative T‐waves were observed in the precordial leads (V1–V4 leads). Additionally, transthoracic echocardiography showed a left ventricular ejection fraction of 30%, an end‐diastolic dimension/end‐systolic dimension of 54/43 mm, diffuse wall‐motion abnormalities, a tricuspid annular plane systolic excursion of 21 mm, mild tricuspid regurgitation, mild mitral regurgitation. On stress myocardial scintigraphy, suspected ischemic findings were observed in the distal region of the left anterior descending artery and the right coronary artery, and coronary angiography (CAG) was performed. The patient was found to have triple vessel disease on the CAG and was scheduled for a CABG. Owing to the patient's low cardiac function, the strategy of on‐pump beating CABG was adopted. In addition, anesthesia management of the patient was considered carefully, and it was decided that drugs that cause minimal circulatory depression should be used as much as possible.

**FIGURE 1:**
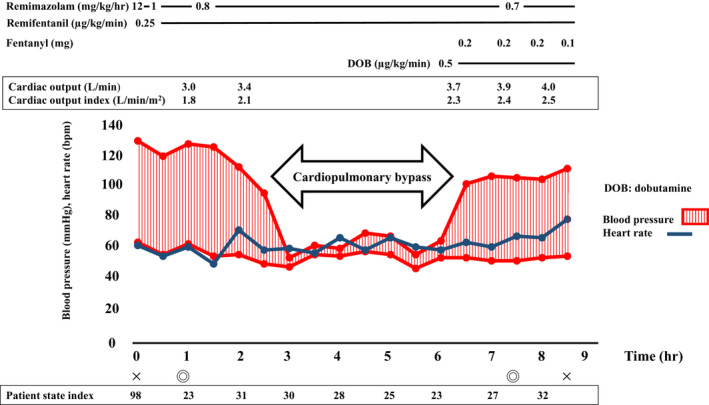
Anesthetic record of the patient.

Anesthesia was induced with remimazolam at 12 mg/kg/hr, remifentanil at 0.3 μg/kg/min, and rocuronium at 50 mg. Anesthesia maintenance was performed with remimazolam at 1 mg/kg/hr, remifentanil at 0.25 μg/kg/min, and rocuronium at 20 mg/hr. An 8‐mm endotracheal tube was used for intubation. Phenylephrine (0.05 mg) was administered as needed for hypotension. During the surgery, monitoring was performed with invasive arterial pressure, peripheral blood oxygen saturation, central venous pressure, patient state index (PSI), regional oxygen saturation, and transesophageal echocardiography. Continuous monitoring of cardiac output was also performed using the FloTrac™ Sensor (Edwards Lifesciences Co., Tokyo, Japan) (Figure 1). From induction to just before the cardiopulmonary bypass (CPB), the cardiac output and cardiac index remained at about 3.0–4.0 L/min and 1.8–2.5 L/min/m^2^, respectively. Stroke volume variation (SVV) remained at about 15%. In addition, administration of the coronary vasodilator nicorandil was started at a rate of 3 mg/hr, and isosorbide dinitrate at a rate of 1 mg/hr from the time of induction. The patient underwent on‐pump beating CABG without any significant hemodynamic instability during the CPB. The grafts used were the left internal thoracic artery (LITA), left radial artery (LRA), and saphenous vein (SV). Five branches (LITA‐left anterior descending coronary artery, aorta‐LRA‐first diagonal branch‐SV‐posterolateral branch‐posterior descending artery) were anastomosed. During separation from the CPB, a continuous infusion of dobutamine at a rate of 0.5 μg/kg/min was administered. The patient's hemodynamic status remained stable without any significant problems even after weaning off from the CPB, and anesthesia was successfully maintained. Transesophageal echocardiography after weaning from the CPB showed mild improvement in wall motion. After separation from the CPB, remimazolam was continuously administered at a rate of 0.7–0.8 mg/kg/hr. We were using the PSI as an indicator of sedation, and PSI values were as shown in Figure 1. Anesthesia time was 8 h and 32 min, surgical time was 7 h and 20 min, and CPB time was 3 h and 43 min. The total amount of fluid administered was 1550 mL, and the amount of bleeding was 1090 mL. The patient received a transfusion of 4 units of red blood cell concentrate and 1000 mL of 5% albumin products. Upon admission to the intensive care unit, the patient was weaned off mechanical ventilation, extubated on postoperative day (POD) 1, and was transferred to a general ward on POD 3. The subsequent course was uneventful, and the patient was discharged on POD 15.

## 3 DISCUSSION

In our present case, the patient showed decreased cardiac function that was thought to be caused by coronary artery stenosis, and hence great attention was paid to the patient's hemodynamics during anesthesia induction. Thus, it was necessary to select anesthetics with minimal circulatory depression during anesthesia induction to avoid circulatory collapse. Careful determination of the dosage of the anesthetics was also required. The same applied to the administration of analgesics. Recently, a short‐acting benzodiazepine called remimazolam was approved for use in Japan.^1^ This drug has a shorter half‐life than midazolam, which is also a benzodiazepine, but has the advantage of a faster onset of action and enables the evaluation of the presence or absence of consciousness using electroencephalography.^3^, ^4^ In our present case, the depth of anesthesia was evaluated by PSI values (Figure 1). The PSI is a parameter of a four‐channel electroencephalography‐derived variable used to assess the depth of anesthesia. A PSI value of 25–50 indicates adequate state of hypnosis, and a value of 100 indicates a fully awake state.^5^ The appropriate depth of anesthesia was maintained during the operation. Another advantage of this drug is that it does not cause vascular pain, unlike propofol.^4^ Before the approval of remimazolam, total intravenous anesthesia with propofol was commonly used for sedation during surgery. However, propofol has a strong circulatory suppression effect,^6^ which makes it difficult to use in patients with decreased cardiac function, indicating a disadvantage. On the other hand, it has been reported that remimazolam causes less circulatory depression, and its use has enabled the maintenance of stable circulatory dynamics even in patients with heart failure.^7^, ^8^, ^9^ In our present case, circulatory management was carried out using FloTrac™ to continuously monitor variables, such as cardiac output and SVV, and both cardiac output and SVV showed stable values without any events of circulatory failure. Our present case indicates that the use of remimazolam is effective in the anesthesia management of patients with decreased cardiac function undergoing cardiac surgery. And a study comparing the incidence of intraoperative awakenings and body movements in the anesthetic management of remimazolam and propofol reported no significant differences in the incidence of these events. This suggests that remimazolam maintains an appropriate depth of anesthesia.^10^ Furthermore, recent reports suggest that compared to propofol, anesthesia management with remimazolam reduces the surgical stress response and the effects of anesthetics on respiratory function, and is superior in reducing anesthesia‐associated side effects.^11^ In addition, remimazolam has been shown to potentially decrease the incidence of postoperative delirium,^12^ and its use is believed to be effective for patients with respiratory abnormalities and older patients at high risk of postoperative delirium.

## 4 CONCLUSION

Remimazolam could be safely used in the anesthetic management of a patient with decreased cardiac function.

## AUTHOR CONTRIBUTIONS

**Shingo Narumi:** Conceptualization; writing – original draft. **Yusuke Ishida:** Conceptualization; writing – original draft; writing – review and editing. **Sae Igarashi:** Writing – review and editing. **Shunya Sekiguchi:** Writing – review and editing. **Aya Kawachi:** Writing – review and editing. **Mikiko Tomino:** Writing – review and editing.

## FUNDING INFORMATION

None.

## CONFLICT OF INTEREST STATEMENT

The authors declare that they have no competing interests associated with this manuscript.

## ETHICS APPROVAL AND CONSENT TO PARTICIPATE

Not applicable.

## CONSENT

Written informed consent was obtained from the patient for publication of this case report and the accompanying images.

## Data Availability

The data that support the findings of this study are available from the corresponding author upon reasonable request.
